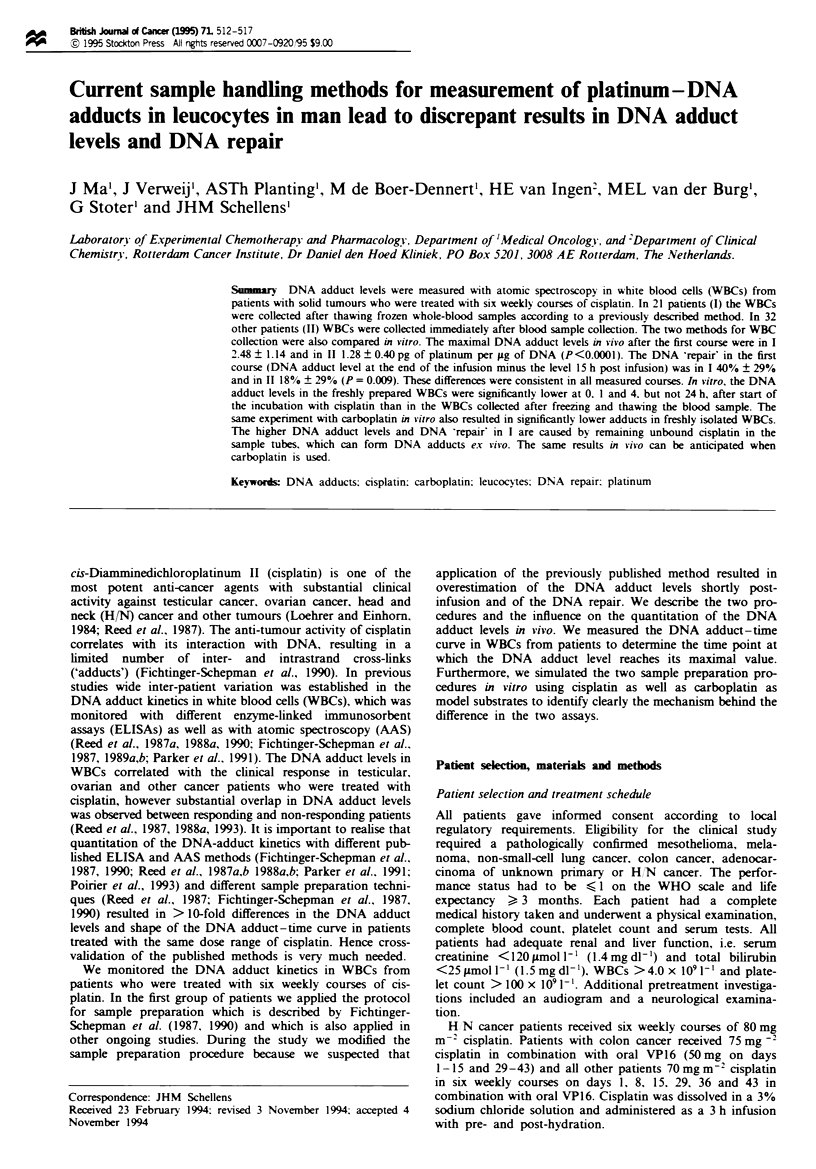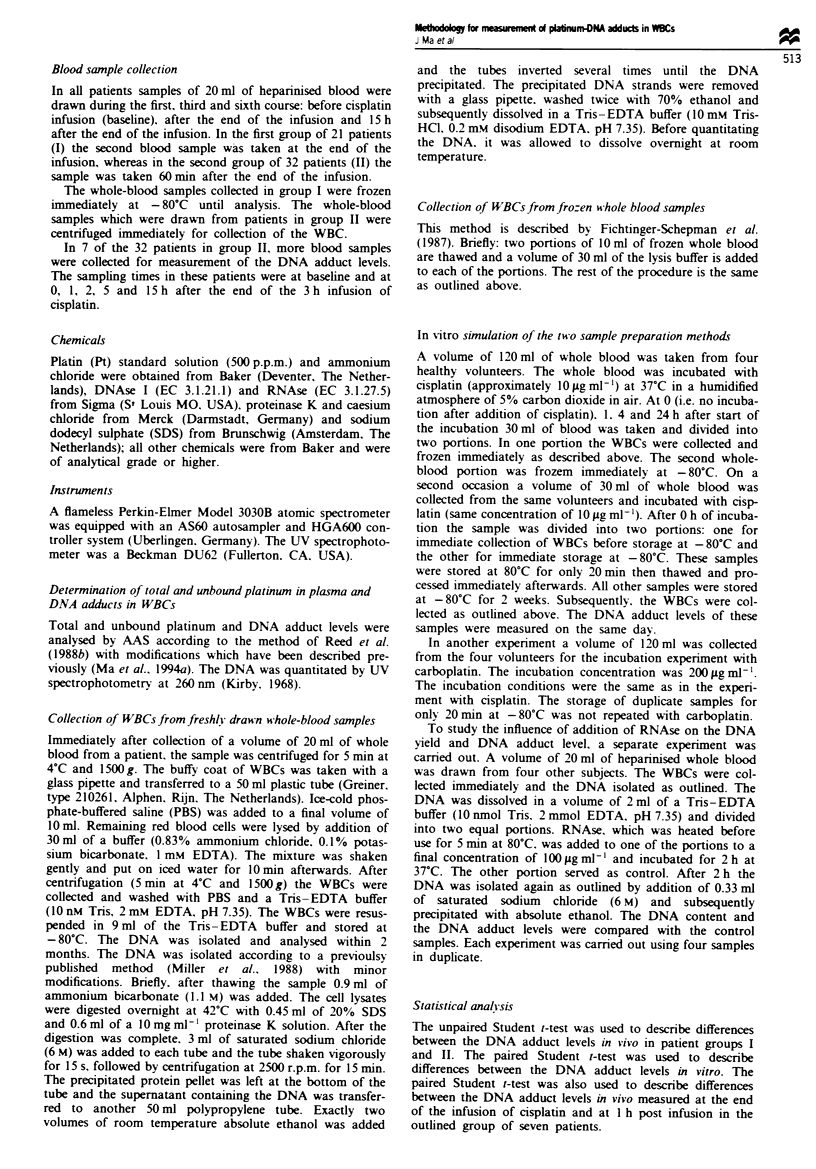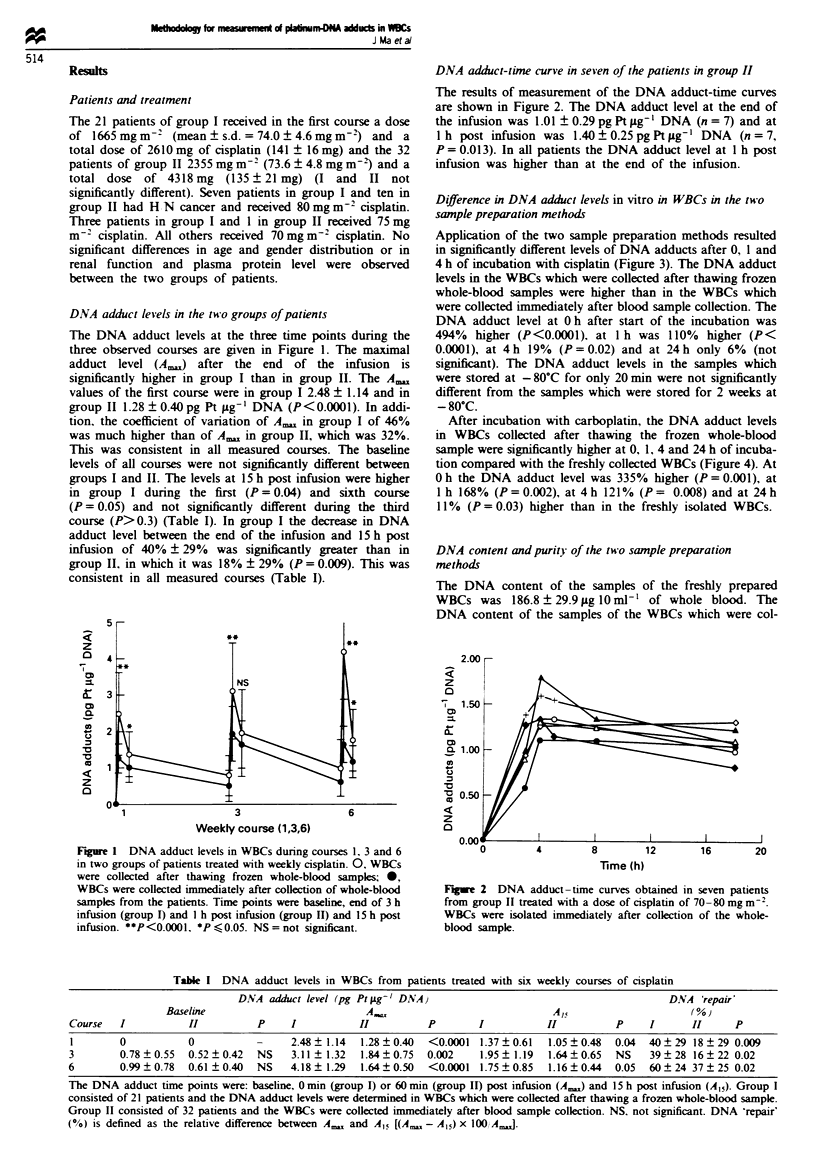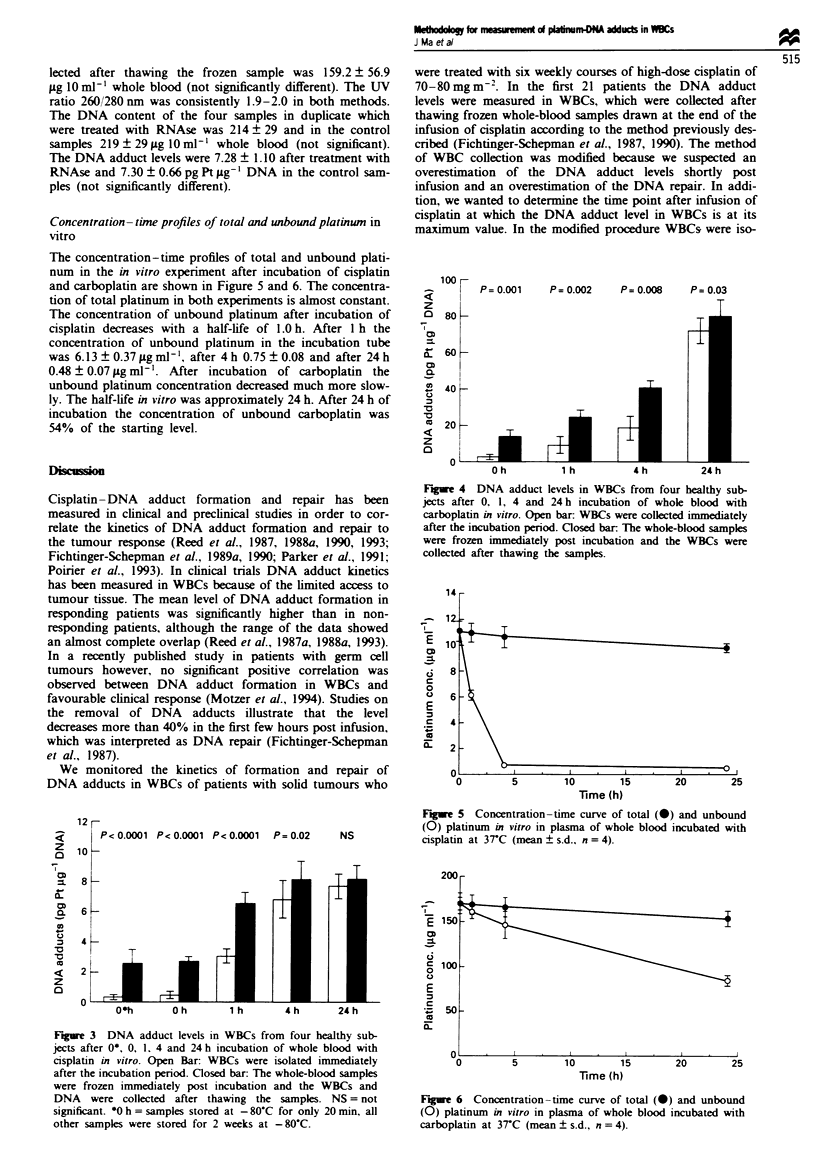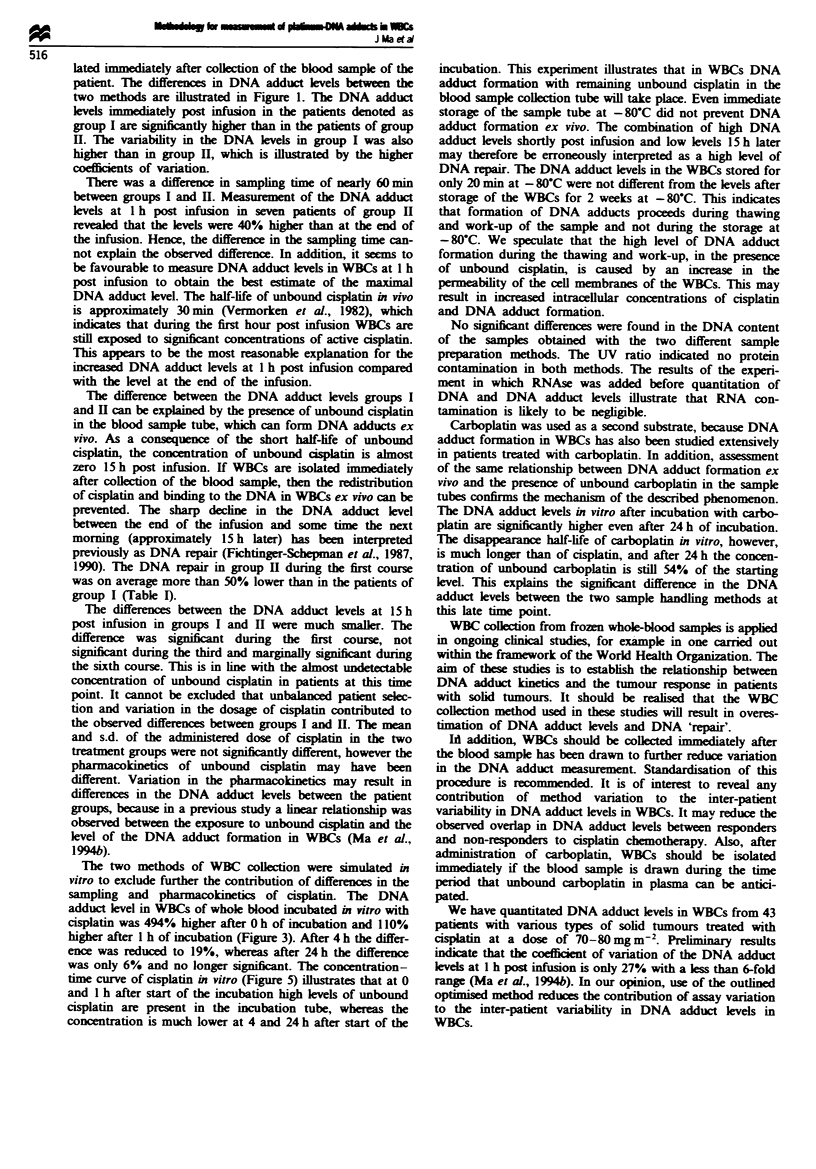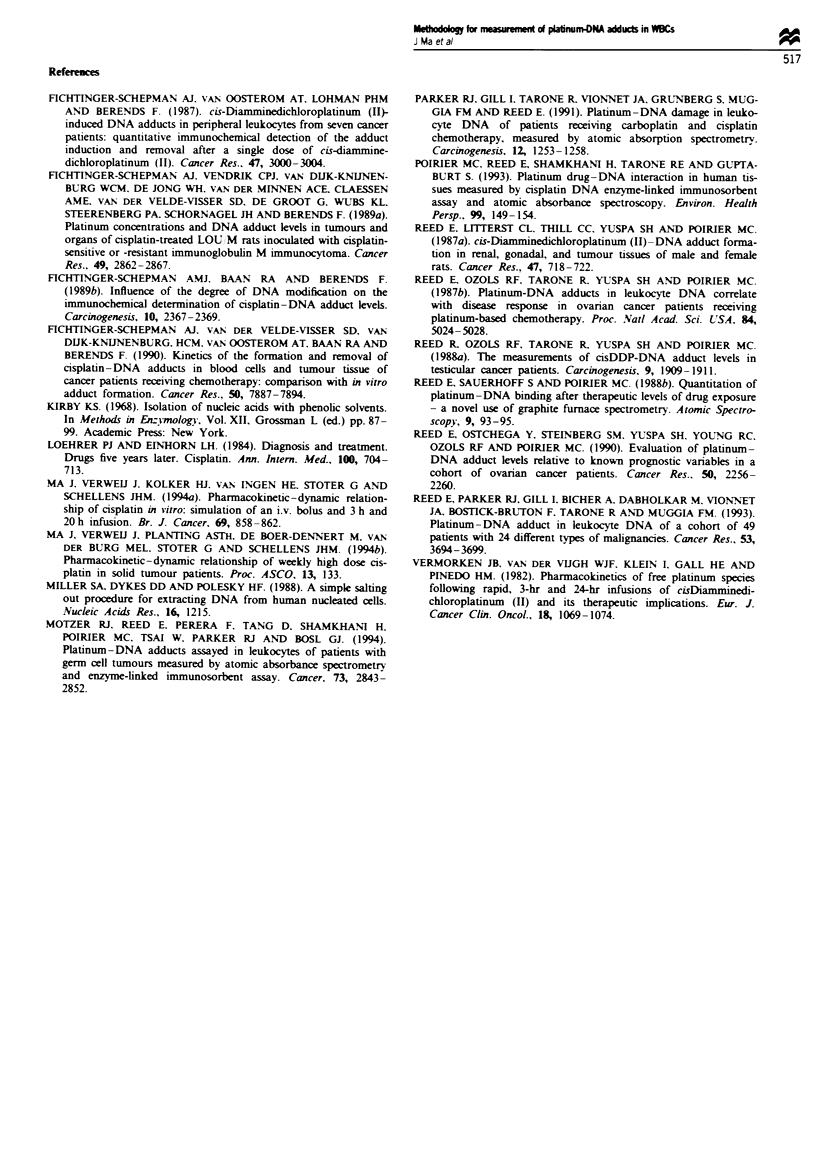# Current sample handling methods for measurement of platinum-DNA adducts in leucocytes in man lead to discrepant results in DNA adduct levels and DNA repair.

**DOI:** 10.1038/bjc.1995.102

**Published:** 1995-03

**Authors:** J. Ma, J. Verweij, A. S. Planting, M. de Boer-Dennert, H. E. van Ingen, M. E. van der Burg, G. Stoter, J. H. Schellens

**Affiliations:** Department of Medical Oncology, Rotterdam Cancer Institute, Dr Daniel den Hoed Kliniek, The Netherlands.

## Abstract

DNA adduct levels were measured with atomic spectroscopy in white blood cells (WBCs) from patients with solid tumours who were treated with six weekly courses of cisplatin. In 21 patients (I) the WBCs were collected after thawing frozen whole-blood samples according to a previously described method. In 32 other patients (II) WBCs were collected immediately after blood sample collection. The two methods for WBC collection were also compared in vitro. The maximal DNA adduct levels in vivo after the first course were in I 2.48 +/- 1.14 and in II 1.28 +/- 0.40 pg of platinum per microgram of DNA (P < 0.0001). The DNA 'repair' in the first course (DNA adduct level at the end of the infusion minus the level 15 h post infusion) was in I 40% +/- 29% and in II 18% +/- 29% (P = 0.009). These differences were consistent in all measured courses. In vitro, the DNA adduct levels in the freshly prepared WBCs were significantly lower at 0, 1 and 4, but not 24 h, after start of the incubation with cisplatin than in the WBCs collected after freezing and thawing the blood sample. The same experiment with carboplatin in vitro also resulted in significantly lower adducts in freshly isolated WBCs. The higher DNA adduct levels and DNA 'repair' in I are caused by remaining unbound cisplatin in the sample tubes, which can form DNA adducts ex vivo. The same results in vivo can be anticipated when carboplatin is used.


British Journal of Cancer (1995) 71, 512–517

© 1995 Stockton Press All rights reserved 0007–0920/95 $9.00

### Current sample handling methods for measurement of platinum–DNA adducts in leucocytes in man lead to discrepant results in DNA adduct levels and DNA repair

J Ma[1], J Verweij[1], ASTh Planting[1], M de Boer-Dennert[1], HE van Ingen[2], MEL van der Burg[1], G Stoter[1] and JHM Schellens[1]

*Laboratory of Experimental Chemotherapy and Pharmacology, Department of [1]Medical Oncology, and [2]Department of Clinical Chemistry, Rotterdam Cancer Institute, Dr Daniel den Hoed Kliniek, PO Box 5201, 3008 AE Rotterdam, The Netherlands.*

**Summary** DNA adduct levels were measured with atomic spectroscopy in white blood cells (WBCs) from patients with solid tumours who were treated with six weekly courses of cisplatin. In 21 patients (I) the WBCs were collected after thawing frozen whole-blood samples according to a previously described method. In 32 other patients (II) WBCs were collected immediately after blood sample collection. The two methods for WBC collection were also compared *in vitro*. The maximal DNA adduct levels *in vivo* after the first course were in I $2.48 \pm 1.14$ and in II $1.28 \pm 0.40$ pg of platinum per μg of DNA ($P<0.0001$). The DNA 'repair' in the first course (DNA adduct level at the end of the infusion minus the level 15 h post infusion) was in I 40% ± 29% and in II 18% ± 29% ($P = 0.009$). These differences were consistent in all measured courses. *In vitro*, the DNA adduct levels in the freshly prepared WBCs were significantly lower at 0, 1 and 4, but not 24 h, after start of the incubation with cisplatin than in the WBCs collected after freezing and thawing the blood sample. The same experiment with carboplatin *in vitro* also resulted in significantly lower adducts in freshly isolated WBCs. The higher DNA adduct levels and DNA 'repair' in I are caused by remaining unbound cisplatin in the sample tubes, which can form DNA adducts *ex vivo*. The same results *in vivo* can be anticipated when carboplatin is used.

**Keywords:** DNA adducts; cisplatin; carboplatin; leucocytes; DNA repair; platinum

*cis*-Diamminedichloroplatinum II (cisplatin) is one of the most potent anti-cancer agents with substantial clinical activity against testicular cancer, ovarian cancer, head and neck (H/N) cancer and other tumours (Loehrer and Einhorn, 1984; Reed *et al*., 1987). The anti-tumour activity of cisplatin correlates with its interaction with DNA, resulting in a limited number of inter- and intrastrand cross-links ('adducts') (Fichtinger-Schepman *et al*., 1990). In previous studies wide inter-patient variation was established in the DNA adduct kinetics in white blood cells (WBCs), which was monitored with different enzyme-linked immunosorbent assays (ELISAs) as well as with atomic spectroscopy (AAS) (Reed *et al*., 1987*a*, 1988*a*, 1990; Fichtinger-Schepman *et al*., 1987, 1989*a*,*b*; Parker *et al*., 1991). The DNA adduct levels in WBCs correlated with the clinical response in testicular, ovarian and other cancer patients who were treated with cisplatin, however substantial overlap in DNA adduct levels was observed between responding and non-responding patients (Reed *et al*., 1987, 1988*a*, 1993). It is important to realise that quantitation of the DNA-adduct kinetics with different published ELISA and AAS methods (Fichtinger-Schepman *et al*., 1987, 1990; Reed *et al*., 1987*a*,*b* 1988*a*,*b*; Parker *et al*., 1991; Poirier *et al*., 1993) and different sample preparation techniques (Reed *et al*., 1987; Fichtinger-Schepman *et al*., 1987, 1990) resulted in >10-fold differences in the DNA adduct levels and shape of the DNA adduct–time curve in patients treated with the same dose range of cisplatin. Hence cross-validation of the published methods is very much needed.

We monitored the DNA adduct kinetics in WBCs from patients who were treated with six weekly courses of cisplatin. In the first group of patients we applied the protocol for sample preparation which is described by Fichtinger-Schepman *et al*. (1987, 1990) and which is also applied in other ongoing studies. During the study we modified the sample preparation procedure because we suspected that application of the previously published method resulted in overestimation of the DNA adduct levels shortly post-infusion and of the DNA repair. We describe the two procedures and the influence on the quantitation of the DNA adduct levels *in vivo*. We measured the DNA adduct–time curve in WBCs from patients to determine the time point at which the DNA adduct level reaches its maximal value. Furthermore, we simulated the two sample preparation procedures *in vitro* using cisplatin as well as carboplatin as model substrates to identify clearly the mechanism behind the difference in the two assays.

### Patient selection, materials and methods

### *Patient selection and treatment schedule*

All patients gave informed consent according to local regulatory requirements. Eligibility for the clinical study required a pathologically confirmed mesothelioma, melanoma, non-small-cell lung cancer, colon cancer, adenocarcinoma of unknown primary or H/N cancer. The performance status had to be ⩽1 on the WHO scale and life expectancy ⩾3 months. Each patient had a complete medical history taken and underwent a physical examination, complete blood count, platelet count and serum tests. All patients had adequate renal and liver function, i.e. serum creatinine $<120\,\mu mol\,l^{-1}$ ($1.4\,mg\,dl^{-1}$) and total bilirubin $<25\,\mu mol\,l^{-1}$ ($1.5\,mg\,dl^{-1}$), WBCs $>4.0\times10^9\,l^{-1}$ and platelet count $>100\times10^9\,l^{-1}$. Additional pretreatment investigations included an audiogram and a neurological examination.

H/N cancer patients received six weekly courses of $80\,mg\,m^{-2}$ cisplatin. Patients with colon cancer received $75\,mg^{-2}$ cisplatin in combination with oral VP16 (50 mg on days 1–15 and 29–43) and all other patients $70\,mg\,m^{-2}$ cisplatin in six weekly courses on days 1, 8, 15, 29, 36 and 43 in combination with oral VP16. Cisplatin was dissolved in a 3% sodium chloride solution and administered as a 3 h infusion with pre- and post-hydration.

Correspondence: JHM Schellens
Received 23 February 1994; revised 3 November 1994; accepted 4 November 1994

*Blood sample collection*

In all patients samples of 20 ml of heparinised blood were drawn during the first, third and sixth course: before cisplatin infusion (baseline), after the end of the infusion and 15 h after the end of the infusion. In the first group of 21 patients (I) the second blood sample was taken at the end of the infusion, whereas in the second group of 32 patients (II) the sample was taken 60 min after the end of the infusion.

The whole-blood samples collected in group I were frozen immediately at −80°C until analysis. The whole-blood samples which were drawn from patients in group II were centrifuged immediately for collection of the WBC.

In 7 of the 32 patients in group II, more blood samples were collected for measurement of the DNA adduct levels. The sampling times in these patients were at baseline and at 0, 1, 2, 5 and 15 h after the end of the 3 h infusion of cisplatin.

*Chemicals*

Platin (Pt) standard solution (500 p.p.m.) and ammonium chloride were obtained from Baker (Deventer, The Netherlands), DNAse I (EC 3.1.21.1) and RNAse (EC 3.1.27.5) from Sigma (St Louis MO, USA), proteinase K and caesium chloride from Merck (Darmstadt, Germany) and sodium dodecyl sulphate (SDS) from Brunschwig (Amsterdam, The Netherlands); all other chemicals were from Baker and were of analytical grade or higher.

*Instruments*

A flameless Perkin-Elmer Model 3030B atomic spectrometer was equipped with an AS60 autosampler and HGA600 controller system (Uberlingen, Germany). The UV spectrophotometer was a Beckman DU62 (Fullerton, CA, USA).

*Determination of total and unbound platinum in plasma and DNA adducts in WBCs*

Total and unbound platinum and DNA adduct levels were analysed by AAS according to the method of Reed *et al.* (1988*b*) with modifications which have been described previously (Ma *et al.*, 1994*a*). The DNA was quantitated by UV spectrophotometry at 260 nm (Kirby, 1968).

*Collection of WBCs from freshly drawn whole-blood samples*

Immediately after collection of a volume of 20 ml of whole blood from a patient, the sample was centrifuged for 5 min at 4°C and 1500 *g*. The buffy coat of WBCs was taken with a glass pipette and transferred to a 50 ml plastic tube (Greiner, type 210261, Alphen, Rijn, The Netherlands). Ice-cold phosphate-buffered saline (PBS) was added to a final volume of 10 ml. Remaining red blood cells were lysed by addition of 30 ml of a buffer (0.83% ammonium chloride, 0.1% potassium bicarbonate, 1 mM EDTA). The mixture was shaken gently and put on iced water for 10 min afterwards. After centrifugation (5 min at 4°C and 1500 *g*) the WBCs were collected and washed with PBS and a Tris–EDTA buffer (10 nM Tris, 2 mM EDTA, pH 7.35). The WBCs were resuspended in 9 ml of the Tris–EDTA buffer and stored at −80°C. The DNA was isolated and analysed within 2 months. The DNA was isolated according to a previoulsy published method (Miller *et al.*, 1988) with minor modifications. Briefly, after thawing the sample 0.9 ml of ammonium bicarbonate (1.1 M) was added. The cell lysates were digested overnight at 42°C with 0.45 ml of 20% SDS and 0.6 ml of a 10 mg $ml^{-1}$ proteinase K solution. After the digestion was complete, 3 ml of saturated sodium chloride (6 M) was added to each tube and the tube shaken vigorously for 15 s, followed by centrifugation at 2500 r.p.m. for 15 min. The precipitated protein pellet was left at the bottom of the tube and the supernatant containing the DNA was transferred to another 50 ml polypropylene tube. Exactly two volumes of room temperature absolute ethanol was added and the tubes inverted several times until the DNA precipitated. The precipitated DNA strands were removed with a glass pipette, washed twice with 70% ethanol and subsequently dissolved in a Tris–EDTA buffer (10 mM Tris-HCl, 0.2 mM disodium EDTA, pH 7.35). Before quantitating the DNA, it was allowed to dissolve overnight at room temperature.

*Collection of WBCs from frozen whole blood samples*

This method is described by Fichtinger-Schepman *et al.* (1987). Briefly: two portions of 10 ml of frozen whole blood are thawed and a volume of 30 ml of the lysis buffer is added to each of the portions. The rest of the procedure is the same as outlined above.

In vitro *simulation of the two sample preparation methods*

A volume of 120 ml of whole blood was taken from four healthy volunteers. The whole blood was incubated with cisplatin (approximately 10 $\mu g\ ml^{-1}$) at 37°C in a humidified atmosphere of 5% carbon dioxide in air. At 0 (i.e. no incubation after addition of cisplatin), 1, 4 and 24 h after start of the incubation 30 ml of blood was taken and divided into two portions. In one portion the WBCs were collected and frozen immediately as described above. The second whole-blood portion was frozem immediately at −80°C. On a second occasion a volume of 30 ml of whole blood was collected from the same volunteers and incubated with cisplatin (same concentration of 10 $\mu g\ ml^{-1}$). After 0 h of incubation the sample was divided into two portions: one for immediate collection of WBCs before storage at −80°C and the other for immediate storage at −80°C. These samples were stored at 80°C for only 20 min then thawed and processed immediately afterwards. All other samples were stored at −80°C for 2 weeks. Subsequently, the WBCs were collected as outlined above. The DNA adduct levels of these samples were measured on the same day.

In another experiment a volume of 120 ml was collected from the four volunteers for the incubation experiment with carboplatin. The incubation concentration was 200 $\mu g\ ml^{-1}$. The incubation conditions were the same as in the experiment with cisplatin. The storage of duplicate samples for only 20 min at −80°C was not repeated with carboplatin.

To study the influence of addition of RNAse on the DNA yield and DNA adduct level, a separate experiment was carried out. A volume of 20 ml of heparinised whole blood was drawn from four other subjects. The WBCs were collected immediately and the DNA isolated as outlined. The DNA was dissolved in a volume of 2 ml of a Tris–EDTA buffer (10 nmol Tris, 2 mmol EDTA, pH 7.35) and divided into two equal portions. RNAse, which was heated before use for 5 min at 80°C, was added to one of the portions to a final concentration of 100 $\mu g\ ml^{-1}$ and incubated for 2 h at 37°C. The other portion served as control. After 2 h the DNA was isolated again as outlined by addition of 0.33 ml of saturated sodium chloride (6 M) and subsequently precipitated with absolute ethanol. The DNA content and the DNA adduct levels were compared with the control samples. Each experiment was carried out using four samples in duplicate.

*Statistical analysis*

The unpaired Student *t*-test was used to describe differences between the DNA adduct levels *in vivo* in patient groups I and II. The paired Student *t*-test was used to describe differences between the DNA adduct levels *in vitro*. The paired Student *t*-test was also used to describe differences between the DNA adduct levels *in vivo* measured at the end of the infusion of cisplatin and at 1 h post infusion in the outlined group of seven patients.

### Results

### *Patients and treatment*

The 21 patients of group I received in the first course a dose of 1665 mg m$^{-2}$ (mean ± s.d. = 74.0 ± 4.6 mg m$^{-2}$) and a total dose of 2610 mg of cisplatin (141 ± 16 mg) and the 32 patients of group II 2355 mg m$^{-2}$ (73.6 ± 4.8 mg m$^{-2}$) and a total dose of 4318 mg (135 ± 21 mg) (I and II not significantly different). Seven patients in group I and ten in group II had H/N cancer and received 80 mg m$^{-2}$ cisplatin. Three patients in group I and 1 in group II received 75 mg m$^{-2}$ cisplatin. All others received 70 mg m$^{-2}$ cisplatin. No significant differences in age and gender distribution or in renal function and plasma protein level were observed between the two groups of patients.

### *DNA adduct levels in the two groups of patients*

The DNA adduct levels at the three time points during the three observed courses are given in Figure 1. The maximal adduct level ($A_{max}$) after the end of the infusion is significantly higher in group I than in group II. The $A_{max}$ values of the first course were in group I 2.48 ± 1.14 and in group II 1.28 ± 0.40 pg Pt µg$^{-1}$ DNA ($P$<0.0001). In addition, the coefficient of variation of $A_{max}$ in group I of 46% was much higher than of $A_{max}$ in group II, which was 32%. This was consistent in all measured courses. The baseline levels of all courses were not significantly different between groups I and II. The levels at 15 h post infusion were higher in group I during the first ($P$ = 0.04) and sixth course ($P$ = 0.05) and not significantly different during the third course ($P$>0.3) (Table I). In group I the decrease in DNA adduct level between the end of the infusion and 15 h post infusion of 40% ± 29% was significantly greater than in group II, in which it was 18% ± 29% ($P$ = 0.009). This was consistent in all measured courses (Table I).

**Figure 1** DNA adduct levels in WBCs during courses 1, 3 and 6 in two groups of patients treated with weekly cisplatin. ○, WBCs were collected after thawing frozen whole-blood samples; ●, WBCs were collected immediately after collection of whole-blood samples from the patients. Time points were baseline, end of 3 h infusion (group I) and 1 h post infusion (group II) and 15 h post infusion. **$P$<0.0001, *$P$ ⩽ 0.05. NS = not significant.

### *DNA adduct-time curve in seven of the patients in group II*

The results of measurement of the DNA adduct-time curves are shown in Figure 2. The DNA adduct level at the end of the infusion was 1.01 ± 0.29 pg Pt µg$^{-1}$ DNA ($n$ = 7) and at 1 h post infusion was 1.40 ± 0.25 pg Pt µg$^{-1}$ DNA ($n$ = 7, $P$ = 0.013). In all patients the DNA adduct level at 1 h post infusion was higher than at the end of the infusion.

### *Difference in DNA adduct levels* in vitro *in WBCs in the two sample preparation methods*

Application of the two sample preparation methods resulted in significantly different levels of DNA adducts after 0, 1 and 4 h of incubation with cisplatin (Figure 3). The DNA adduct levels in the WBCs which were collected after thawing frozen whole-blood samples were higher than in the WBCs which were collected immediately after blood sample collection. The DNA adduct level at 0 h after start of the incubation was 494% higher ($P$<0.0001), at 1 h was 110% higher ($P$< 0.0001), at 4 h 19% ($P$ = 0.02) and at 24 h only 6% (not significant). The DNA adduct levels in the samples which were stored at −80°C for only 20 min were not significantly different from the samples which were stored for 2 weeks at −80°C.

After incubation with carboplatin, the DNA adduct levels in WBCs collected after thawing the frozen whole-blood sample were significantly higher at 0, 1, 4 and 24 h of incubation compared with the freshly collected WBCs (Figure 4). At 0 h the DNA adduct level was 335% higher ($P$ = 0.001), at 1 h 168% ($P$ = 0.002), at 4 h 121% ($P$ = 0.008) and at 24 h 11% ($P$ = 0.03) higher than in the freshly isolated WBCs.

### *DNA content and purity of the two sample preparation methods*

The DNA content of the samples of the freshly prepared WBCs was 186.8 ± 29.9 µg 10 ml$^{-1}$ of whole blood. The DNA content of the samples of the WBCs which were col-

**Figure 2** DNA adduct-time curves obtained in seven patients from group II treated with a dose of cisplatin of 70–80 mg m$^{-2}$. WBCs were isolated immediately after collection of the whole-blood sample.

**Table I** DNA adduct levels in WBCs from patients treated with six weekly courses of cisplatin

| | DNA adduct level (pg Pt µg$^{-1}$ DNA) | | | | | | | | | DNA 'repair' (%) | | |
|---|---|---|---|---|---|---|---|---|---|---|---|---|
| | Baseline | | | $A_{max}$ | | | $A_{15}$ | | | | | |
| Course | I | II | P | I | II | P | I | II | P | I | II | P |
| 1 | 0 | 0 | – | 2.48 ± 1.14 | 1.28 ± 0.40 | <0.0001 | 1.37 ± 0.61 | 1.05 ± 0.48 | 0.04 | 40 ± 29 | 18 ± 29 | 0.009 |
| 3 | 0.78 ± 0.55 | 0.52 ± 0.42 | NS | 3.11 ± 1.32 | 1.84 ± 0.75 | 0.002 | 1.95 ± 1.19 | 1.64 ± 0.65 | NS | 39 ± 28 | 16 ± 22 | 0.02 |
| 6 | 0.99 ± 0.78 | 0.61 ± 0.40 | NS | 4.18 ± 1.29 | 1.64 ± 0.50 | <0.0001 | 1.75 ± 0.85 | 1.16 ± 0.44 | 0.05 | 60 ± 24 | 37 ± 25 | 0.02 |

The DNA adduct time points were: baseline, 0 min (group I) or 60 min (group II) post infusion ($A_{max}$) and 15 h post infusion ($A_{15}$). Group I consisted of 21 patients and the DNA adduct levels were determined in WBCs which were collected after thawing a frozen whole-blood sample. Group II consisted of 32 patients and the WBCs were collected immediately after blood sample collection. NS, not significant. DNA 'repair' (%) is defined as the relative difference between $A_{max}$ and $A_{15}$ [($A_{max}$ − $A_{15}$) × 100/$A_{max}$].

lected after thawing the frozen sample was 159.2 ± 56.9 μg 10 ml$^{-1}$ whole blood (not significantly different). The UV ratio 260/280 nm was consistently 1.9–2.0 in both methods. The DNA content of the four samples in duplicate which were treated with RNAse was 214 ± 29 and in the control samples 219 ± 29 μg 10 ml$^{-1}$ whole blood (not significant). The DNA adduct levels were 7.28 ± 1.10 after treatment with RNAse and 7.30 ± 0.66 pg Pt μg$^{-1}$ DNA in the control samples (not significantly different).

*Concentration–time profiles of total and unbound platinum* in vitro

The concentration–time profiles of total and unbound platinum in the *in vitro* experiment after incubation of cisplatin and carboplatin are shown in Figure 5 and 6. The concentration of total platinum in both experiments is almost constant. The concentration of unbound platinum after incubation of cisplatin decreases with a half-life of 1.0 h. After 1 h the concentration of unbound platinum in the incubation tube was 6.13 ± 0.37 μg ml$^{-1}$, after 4 h 0.75 ± 0.08 and after 24 h 0.48 ± 0.07 μg ml$^{-1}$. After incubation of carboplatin the unbound platinum concentration decreased much more slowly. The half-life *in vitro* was approximately 24 h. After 24 h of incubation the concentration of unbound carboplatin was 54% of the starting level.

### Discussion

Cisplatin–DNA adduct formation and repair has been measured in clinical and preclinical studies in order to correlate the kinetics of DNA adduct formation and repair to the tumour response (Reed *et al.*, 1987, 1988*a*, 1990, 1993; Fichtinger-Schepman *et al.*, 1989*a*, 1990; Parker *et al.*, 1991; Poirier *et al.*, 1993). In clinical trials DNA adduct kinetics has been measured in WBCs because of the limited access to tumour tissue. The mean level of DNA adduct formation in responding patients was significantly higher than in non-responding patients, although the range of the data showed an almost complete overlap (Reed *et al.*, 1987*a*, 1988*a*, 1993). In a recently published study in patients with germ cell tumours however, no significant positive correlation was observed between DNA adduct formation in WBCs and favourable clinical response (Motzer *et al.*, 1994). Studies on the removal of DNA adducts illustrate that the level decreases more than 40% in the first few hours post infusion, which was interpreted as DNA repair (Fichtinger-Schepman *et al.*, 1987).

We monitored the kinetics of formation and repair of DNA adducts in WBCs of patients with solid tumours who were treated with six weekly courses of high-dose cisplatin of 70–80 mg m$^{-2}$. In the first 21 patients the DNA adduct levels were measured in WBCs, which were collected after thawing frozen whole-blood samples drawn at the end of the infusion of cisplatin according to the method previously described (Fichtinger-Schepman *et al.*, 1987, 1990). The method of WBC collection was modified because we suspected an overestimation of the DNA adduct levels shortly post infusion and an overestimation of the DNA repair. In addition, we wanted to determine the time point after infusion of cisplatin at which the DNA adduct level in WBCs is at its maximum value. In the modified procedure WBCs were iso-

**Figure 3** DNA adduct levels in WBCs from four healthy subjects after 0*, 0, 1, 4 and 24 h incubation of whole blood with cisplatin *in vitro*. Open Bar: WBCs were isolated immediately after the incubation period. Closed bar: The whole-blood samples were frozen immediately post incubation and the WBCs and DNA were collected after thawing the samples. NS = not significant. *0 h = samples stored at −80°C for only 20 min, all other samples were stored for 2 weeks at −80°C.

**Figure 4** DNA adduct levels in WBCs from four healthy subjects after 0, 1, 4 and 24 h incubation of whole blood with carboplatin *in vitro*. Open bar: WBCs were collected immediately after the incubation period. Closed bar: The whole-blood samples were frozen immediately post incubation and the WBCs were collected after thawing the samples.

**Figure 5** Concentration–time curve of total (●) and unbound (○) platinum *in vitro* in plasma of whole blood incubated with cisplatin at 37°C (mean ± s.d., $n = 4$).

**Figure 6** Concentration–time curve of total (●) and unbound (○) platinum *in vitro* in plasma of whole blood incubated with carboplatin at 37°C (mean ± s.d., $n = 4$).

lated immediately after collection of the blood sample of the patient. The differences in DNA adduct levels between the two methods are illustrated in Figure 1. The DNA adduct levels immediately post infusion in the patients denoted as group I are significantly higher than in the patients of group II. The variability in the DNA levels in group I was also higher than in group II, which is illustrated by the higher coefficients of variation.

There was a difference in sampling time of nearly 60 min between groups I and II. Measurement of the DNA adduct levels at 1 h post infusion in seven patients of group II revealed that the levels were 40% higher than at the end of the infusion. Hence, the difference in the sampling time cannot explain the observed difference. In addition, it seems to be favourable to measure DNA adduct levels in WBCs at 1 h post infusion to obtain the best estimate of the maximal DNA adduct level. The half-life of unbound cisplatin *in vivo* is approximately 30 min (Vermorken *et al.*, 1982), which indicates that during the first hour post infusion WBCs are still exposed to significant concentrations of active cisplatin. This appears to be the most reasonable explanation for the increased DNA adduct levels at 1 h post infusion compared with the level at the end of the infusion.

The difference between the DNA adduct levels groups I and II can be explained by the presence of unbound cisplatin in the blood sample tube, which can form DNA adducts *ex vivo*. As a consequence of the short half-life of unbound cisplatin, the concentration of unbound cisplatin is almost zero 15 h post infusion. If WBCs are isolated immediately after collection of the blood sample, then the redistribution of cisplatin and binding to the DNA in WBCs *ex vivo* can be prevented. The sharp decline in the DNA adduct level between the end of the infusion and some time the next morning (approximately 15 h later) has been interpreted previously as DNA repair (Fichtinger-Schepman *et al.*, 1987, 1990). The DNA repair in group II during the first course was on average more than 50% lower than in the patients of group I (Table I).

The differences between the DNA adduct levels at 15 h post infusion in groups I and II were much smaller. The difference was significant during the first course, not significant during the third and marginally significant during the sixth course. This is in line with the almost undetectable concentration of unbound cisplatin in patients at this time point. It cannot be excluded that unbalanced patient selection and variation in the dosage of cisplatin contributed to the observed differences between groups I and II. The mean and s.d. of the administered dose of cisplatin in the two treatment groups were not significantly different, however the pharmacokinetics of unbound cisplatin may have been different. Variation in the pharmacokinetics may result in differences in the DNA adduct levels between the patient groups, because in a previous study a linear relationship was observed between the exposure to unbound cisplatin and the level of the DNA adduct formation in WBCs (Ma *et al.*, 1994*b*).

The two methods of WBC collection were simulated *in vitro* to exclude further the contribution of differences in the sampling and pharmacokinetics of cisplatin. The DNA adduct level in WBCs of whole blood incubated *in vitro* with cisplatin was 494% higher after 0 h of incubation and 110% higher after 1 h of incubation (Figure 3). After 4 h the difference was reduced to 19%, whereas after 24 h the difference was only 6% and no longer significant. The concentration–time curve of cisplatin *in vitro* (Figure 5) illustrates that at 0 and 1 h after start of the incubation high levels of unbound cisplatin are present in the incubation tube, whereas the concentration is much lower at 4 and 24 h after start of the incubation. This experiment illustrates that in WBCs DNA adduct formation with remaining unbound cisplatin in the blood sample collection tube will take place. Even immediate storage of the sample tube at −80°C did not prevent DNA adduct formation *ex vivo*. The combination of high DNA adduct levels shortly post infusion and low levels 15 h later may therefore be erroneously interpreted as a high level of DNA repair. The DNA adduct levels in the WBCs stored for only 20 min at −80°C were not different from the levels after storage of the WBCs for 2 weeks at −80°C. This indicates that formation of DNA adducts proceeds during thawing and work-up of the sample and not during the storage at −80°C. We speculate that the high level of DNA adduct formation during the thawing and work-up, in the presence of unbound cisplatin, is caused by an increase in the permeability of the cell membranes of the WBCs. This may result in increased intracellular concentrations of cisplatin and DNA adduct formation.

No significant differences were found in the DNA content of the samples obtained with the two different sample preparation methods. The UV ratio indicated no protein contamination in both methods. The results of the experiment in which RNAse was added before quantitation of DNA and DNA adduct levels illustrate that RNA contamination is likely to be negligible.

Carboplatin was used as a second substrate, because DNA adduct formation in WBCs has also been studied extensively in patients treated with carboplatin. In addition, assessment of the same relationship between DNA adduct formation *ex vivo* and the presence of unbound carboplatin in the sample tubes confirms the mechanism of the described phenomenon. The DNA adduct levels *in vitro* after incubation with carboplatin are significantly higher even after 24 h of incubation. The disappearance half-life of carboplatin *in vitro*, however, is much longer than of cisplatin, and after 24 h the concentration of unbound carboplatin is still 54% of the starting level. This explains the significant difference in the DNA adduct levels between the two sample handling methods at this late time point.

WBC collection from frozen whole-blood samples is applied in ongoing clinical studies, for example in one carried out within the framework of the World Health Organization. The aim of these studies is to establish the relationship between DNA adduct kinetics and the tumour response in patients with solid tumours. It should be realised that the WBC collection method used in these studies will result in overestimation of DNA adduct levels and DNA 'repair'.

In addition, WBCs should be collected immediately after the blood sample has been drawn to further reduce variation in the DNA adduct measurement. Standardisation of this procedure is recommended. It is of interest to reveal any contribution of method variation to the inter-patient variability in DNA adduct levels in WBCs. It may reduce the observed overlap in DNA adduct levels between responders and non-responders to cisplatin chemotherapy. Also, after administration of carboplatin, WBCs should be isolated immediately if the blood sample is drawn during the time period that unbound carboplatin in plasma can be anticipated.

We have quantitated DNA adduct levels in WBCs from 43 patients with various types of solid tumours treated with cisplatin at a dose of 70–80 mg $m^{-2}$. Preliminary results indicate that the coefficient of variation of the DNA adduct levels at 1 h post infusion is only 27% with a less than 6-fold range (Ma *et al.*, 1994*b*). In our opinion, use of the outlined optimised method reduces the contribution of assay variation to the inter-patient variability in DNA adduct levels in WBCs.

### References

FICHTINGER-SCHEPMAN AJ, VAN OOSTEROM AT, LOHMAN PHM AND BERENDS F. (1987). *cis*-Diamminedichloroplatinum (II)-induced DNA adducts in peripheral leukocytes from seven cancer patients: quantitative immunochemical detection of the adduct induction and removal after a single dose of *cis*-diamminedichloroplatinum (II). *Cancer Res.*, **47,** 3000–3004.

FICHTINGER-SCHEPMAN AJ, VENDRIK CPJ, VAN DIJK-KNIJNENBURG WCM, DE JONG WH, VAN DER MINNEN ACE, CLAESSEN AME, VAN DER VELDE-VISSER SD, DE GROOT G, WUBS KL, STEERENBERG PA, SCHORNAGEL JH AND BERENDS F. (1989*a*). Platinum concentrations and DNA adduct levels in tumours and organs of cisplatin-treated LOU/M rats inoculated with cisplatin-sensitive or -resistant immunoglobulin M immunocytoma. *Cancer Res.*, **49,** 2862–2867.

FICHTINGER-SCHEPMAN AMJ, BAAN RA AND BERENDS F. (1989*b*). Influence of the degree of DNA modification on the immunochemical determination of cisplatin–DNA adduct levels. *Carcinogenesis*, **10,** 2367–2369.

FICHTINGER-SCHEPMAN AJ, VAN DER VELDE-VISSER SD, VAN DIJK-KNIJNENBURG, HCM, VAN OOSTEROM AT, BAAN RA AND BERENDS F. (1990). Kinetics of the formation and removal of cisplatin–DNA adducts in blood cells and tumour tissue of cancer patients receiving chemotherapy: comparison with *in vitro* adduct formation. *Cancer Res.*, **50,** 7887–7894.

KIRBY KS. (1968). Isolation of nucleic acids with phenolic solvents. In *Methods in Enzymology*, Vol. XII, Grossman L (ed.) pp. 87–99. Academic Press: New York.

LOEHRER PJ AND EINHORN LH. (1984). Diagnosis and treatment. Drugs five years later. Cisplatin. *Ann. Intern. Med.*, **100,** 704–713.

MA J, VERWEIJ J, KOLKER HJ, VAN INGEN HE, STOTER G AND SCHELLENS JHM. (1994*a*). Pharmacokinetic–dynamic relationship of cisplatin *in vitro*: simulation of an i.v. bolus and 3 h and 20 h infusion. *Br. J. Cancer*, **69,** 858–862.

MA J, VERWEIJ J, PLANTING ASTH, DE BOER-DENNERT M, VAN DER BURG MEL, STOTER G AND SCHELLENS JHM. (1994*b*). Pharmacokinetic–dynamic relationship of weekly high dose cisplatin in solid tumour patients. *Proc. ASCO*, **13,** 133.

MILLER SA, DYKES DD AND POLESKY HF. (1988). A simple salting out procedure for extracting DNA from human nucleated cells. *Nucleic Acids Res.*, **16,** 1215.

MOTZER RJ, REED E, PERERA F, TANG D, SHAMKHANI H, POIRIER MC, TSAI W, PARKER RJ AND BOSL GJ. (1994). Platinum–DNA adducts assayed in leukocytes of patients with germ cell tumours measured by atomic absorbance spectrometry and enzyme-linked immunosorbent assay. *Cancer*, **73,** 2843–2852.

PARKER RJ, GILL I, TARONE R, VIONNET JA, GRUNBERG S, MUGGIA FM AND REED E. (1991). Platinum–DNA damage in leukocyte DNA of patients receiving carboplatin and cisplatin chemotherapy, measured by atomic absorption spectrometry. *Carcinogenesis*, **12,** 1253–1258.

POIRIER MC, REED E, SHAMKHANI H, TARONE RE AND GUPTA-BURT S. (1993). Platinum drug–DNA interaction in human tissues measured by cisplatin DNA enzyme-linked immunosorbent assay and atomic absorbance spectroscopy. *Environ. Health Persp.*, **99,** 149–154.

REED E, LITTERST CL, THILL CC, YUSPA SH AND POIRIER MC. (1987*a*). *cis*-Diamminedichloroplatinum (II)–DNA adduct formation in renal, gonadal, and tumour tissues of male and female rats. *Cancer Res.*, **47,** 718–722.

REED E, OZOLS RF, TARONE R, YUSPA SH AND POIRIER MC. (1987*b*). Platinum-DNA adducts in leukocyte DNA correlate with disease response in ovarian cancer patients receiving platinum-based chemotherapy. *Proc. Natl Acad. Sci. USA*, **84,** 5024–5028.

REED R, OZOLS RF, TARONE R, YUSPA SH AND POIRIER MC. (1988*a*). The measurements of cisDDP-DNA adduct levels in testicular cancer patients. *Carcinogenesis*, **9,** 1909–1911.

REED E, SAUERHOFF S AND POIRIER MC. (1988*b*). Quantitation of platinum–DNA binding after therapeutic levels of drug exposure – a novel use of graphite furnace spectrometry. *Atomic Spectroscopy*, **9,** 93–95.

REED E, OSTCHEGA Y, STEINBERG SM, YUSPA SH, YOUNG RC, OZOLS RF AND POIRIER MC. (1990). Evaluation of platinum–DNA adduct levels relative to known prognostic variables in a cohort of ovarian cancer patients. *Cancer Res.*, **50,** 2256–2260.

REED E, PARKER RJ, GILL I, BICHER A, DABHOLKAR M, VIONNET JA, BOSTICK-BRUTON F, TARONE R AND MUGGIA FM. (1993). Platinum–DNA adduct in leukocyte DNA of a cohort of 49 patients with 24 different types of malignancies. *Cancer Res.*, **53,** 3694–3699.

VERMORKEN JB, VAN DER VIJGH WJF, KLEIN I, GALL HE AND PINEDO HM. (1982). Pharmacokinetics of free platinum species following rapid, 3-hr and 24-hr infusions of *cis*Diamminedichloroplatinum (II) and its therapeutic implications. *Eur. J. Cancer Clin. Oncol.*, **18,** 1069–1074.